# Isolation, Amino Acid Sequences, and Plausible Functions of the Galacturonic Acid-Binding Egg Lectin of the Sea Hare *Aplysia kurodai*

**DOI:** 10.3390/md15060161

**Published:** 2017-06-02

**Authors:** Shoko Motohashi, Mitsuru Jimbo, Tomohiro Naito, Takefumi Suzuki, Ryuichi Sakai, Hisao Kamiya

**Affiliations:** 1 Department of Marine Biosciences, School of Marine Biosciences, Kitasato University,1-15-1, Minami-ku, Kitasato, Sagamihara, Kanagawa 252-0373, Japan; Shoko.f00176@gmail.com (S.M.); fm12136m@st.kitasato-u.ac.jp (T.N.); dondoko.mon.123@docomo.ne.jp (T.S.); hkami@jcom.home.ne.jp (H.K.); 2 Faculty of Fisheries Sciences, Hokkaido University, Hakodate 041-8611, Japan; ryu.sakai@fish.hokudai.ac.jp

**Keywords:** *Aplysia kurodai*, egg lectin, embryonic development, galacturonic acid

## Abstract

Egg lectins occur in a variety of animals ranging from mollusks to vertebrates. A few examples of molluscan egg lectins have been reported, including that of the sea hare *Aplysia kurodai*; however, their biological functions in the egg remain unclarified. We report the isolation, determination of primary structure, and possible functions of *A.* *kurodai* lectin (AKL) from the egg mass of *A. kurodai*. We obtained AKL as an inseparable mixture of isoproteins with a relative molecular mass of approximately 32 kDa by affinity purification. The hemagglutinating activity of AKL against rabbit erythrocytes was inhibited most potently by galacturonic acid and moderately by xylose. Nucleotide sequencing of corresponding cDNA obtained by rapid amplification of cDNA ends (RACE) allowed us to deduce complete amino acid sequences. The mature polypeptides consisted of 218- or 219-amino acids with three repeated domains. The amino acid sequence had similarities to hypothetical proteins of *Aplysia* spp., or domain DUF3011 of uncharacterized bacterial proteins. AKL is the first member of the DUF3011 family whose function, carbohydrate recognition, was revealed. Treatment of the egg with galacturonic acid, an AKL sugar inhibitor, resulted in deformation of the veliger larvae, suggesting that AKL is involved in organogenesis in the developmental stage of *A. kurodai*.

## 1. Introduction

Lectins are a large group of proteins, except for enzymes and immunoglobulins, that bind to sugars. Lectins are classified primarily by their amino acid sequences and by sugar selectivity; for example, calcium-dependent C-type lectin, β-galactoside binding lectin, galectin, and so on. However, many lectins do not fit with the above categories because of their sequence divergence. This means that there are so many proteins that can be called “lectin” with novel amino acid sequence and domain architectures and therefore their biological functions are of great interest. Animal lectins are involved in biological processes including chemical defense, tumor cell growth, synaptic receptor modulation, mitogenic processes, biomineralization, symbiosis, and development [1,2,3,4,5,6]. In the case of egg-borne lectins, the possible functions have been studied in relation to important biological phenomena. Numerous examples are reported in a wide variety of animals, such as fish [7,8,9], amphibia [1,10], echinoderms [11], and mollusks [12]. The function of egg lectins was often speculated to be development-related based on their distribution and dynamics. For example, *Xenopus laevis* lectin, a 43–45 kDa galactose-binding lectin, is localized in a vesicle beneath the egg surface [13], but, after fertilization, the lectin is released into the extracellular matrix and distributed around the blastopore and the roof of the blastocoel [14].

The egg of the steelhead trout *Onchorhynchus mykiss* contains three isolectins (STL-1, -2 and -3) [15], which are localized on cortical granules of the egg [16]. STL-2, and -3 decrease after hatching, while STL-1 temporarily increases after hatching and then decreases [15]. In *Gallus gallus*, a galactose-binding lectin was detected at a different stage [17]; at the early stage, the lectin was distributed most at the epiblast and hypoblast in the region of the primitive streak. During gastrulation, strong immunoreactivity to the lectin was detected in migrating cells and the mesoblast, and at the ten somite stage, the lectin was distributed at the neural tube and the presumptive cardiac region [17]. Interestingly, the addition of a horseshoe crab lectin resulted in enlargement of the heart of a chick embryo by increasing the number of heart cells [18]. These observations clearly indicate that lectins play important roles in early development.

The function of sea hare egg lectin, however, remains unclarified, due mainly to difficulty in obtaining pure functional proteins for chemical and biological research. The lectin was first obtained from the sea hare *Aplysia kurodai* by affinity chromatography on d-galacturonic acid (GalU)-Sepharose 6B, and was reported to be a hexamer of a 13 kDa subunit [12]. Three cytotoxic egg lectins with molecular masses of 16 and 32 kDa were later reported from the same species [19,20]. However, no amino acid sequence was reported in either case [12,19,20]. GalU-binding lectin was also purified from the gonad of *A. depilans*, and was found to bind I antigen [21,22]. Thus far, no further structural information besides the molecular masses of these lectins is available. We therefore reinvestigated the structure and function of the lectin of the egg mass of *A. kurodai*. Herein, we report the isolation, chemo-physical characterization, and cDNA cloning of *A. kurodai* lectin (AKL). We also report the effect of a sugar inhibitor of AKL on embryonic development, and discuss its possible functions.

## 2. Results

### 2.1. Isolation of Egg Lectins

The egg mass extract showed strong hemagglutinating activity against rabbit erythrocytes. The activity was not Ca^2+^ dependent, but was strongly inhibited by d-galacturonic acid among the sugars tested, as reported previously [12].

**Figure 1:**
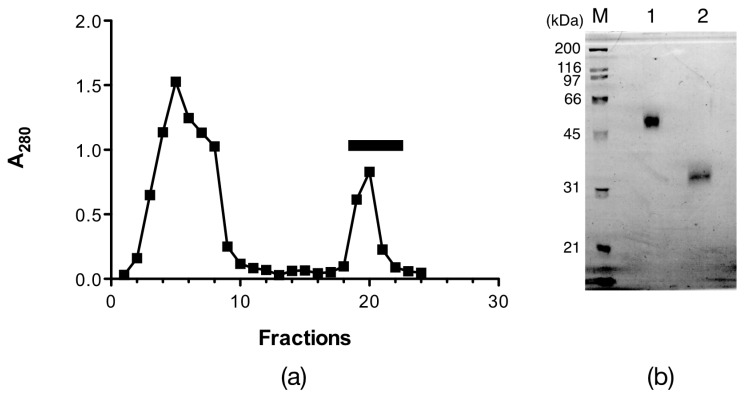
Affinity chromatography of *Aplysia kurodai* lectin (AKL). (**a**) affinity purification of AKL. The crude egg mass extract was applied to a d-galactose-Sepharose 6B affinity column. Unbound proteins were eluted by washing with 20 mM Tris-HCl, pH 8.0, 0.15 M NaCl (the first peak), and the bound lectin was eluted by 0.2 M d-galactose (the second peak, bar); (**b**) sodium dodecyl sulfate-polyacrylamide gel electrophoresis (SDS-PAGE) of purified AKL under non-reducing conditions (lane 1), reducing conditions (lane 2), and molecular standards (M).

The egg lectin was affinity-purified by d-galactose Sepharose 6B, where the flow-through fractions did not have any hemagglutinating activity, while one peak appeared by adding 0.2 M d-galactose, which showed potent hemagglutinating activity after dialysis (Figure 1a). The eluate had a single band with an apparent molecular mass of 33.9 kDa under reducing conditions and 50 kDa under non-reducing conditions by sodium dodecyl sulfate-polyacrylamide gel electrophoresis (SDS-PAGE) (Figure 1b). However, the *N*-terminal amino acid sequence for the 33.9 kDa band indicated that the purified lectin was a mixture of two or more proteins (Table 1 SDS). Although further purifications by ion-exchange chromatography and chromatofocusing were attempted, only a broad peak was obtained. Thus, this affinity-purified peak was denoted as AKL and was used in further experiments.

**Figure 2:**
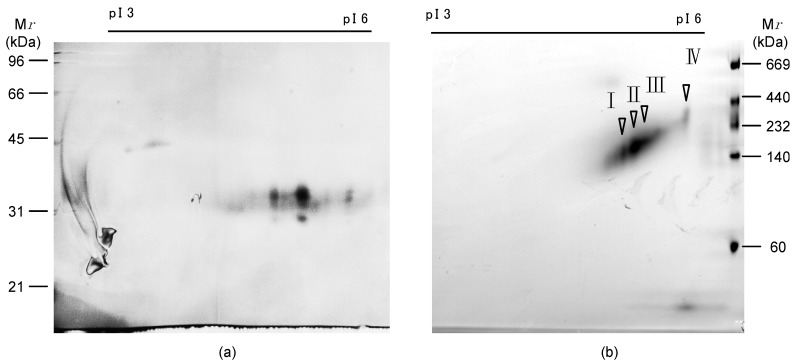
Two-dimensional electrophoresis (2DE) of *Aplysia kurodai* lectin (AKL). 2DE of purified AKL under reducing conditions (**a**), or non-reducing conditions (**b**), where pI and M*r* indicate isoelectric point and relative molecular mass, respectively. I, II, III and IV indicate the spots analyzed by protein sequencer.

On denaturing two-dimensional electrophoresis (2-DE), the pI of AKL ranged from 4.5 to 5.4. At each pI, three spots with different molecular masses of 30.5, 32.7 and 33.9 kDa were observed (Figure 2a). On the native 2-DE, the pI of these spots ranged widely from 4.9 to 5.5, and the relative molecular mass (*Mr*) ranged widely from 131 to 287 kDa (Figure 2b). The major spot was at pI 5.1 and *Mr* 147 kDa. These results indicated that AKL is a complex isolectin, and that the protomers assemble to form multimeric lectins.

### 2.2. The N-Terminal and Partial Amino Acid Sequence of AKL

In Edman degradation of AKL obtained as a single band by SDS-PAGE, two sets of amino acids were detected at each residue from the *N*-terminal up to the 10th residues (Table 1 SDS), showing the complex nature of AKL as a mixture of isoproteins. Thus, the lectin was separated by native two-dimensional electrophoresis (2DE) and sequenced. Four spots on 2DE gave sequences from a single peptide, while the spots on denaturing 2DE still gave a signal for a mixture. The sequences of each spot from the native 2DE (Figure 2b) were very similar to each other (Table 1). The amino acid sequences coincided with one of the amino acid sequences of AKL separated by SDS-PAGE.

To determine the internal amino acid sequences of AKL, peptides obtained from in-gel digestion of AKL by trypsin were analyzed by matrix assisted laser desorption ionization-time of flight mass spectrometry (MALDI-TOF MS). The obtained sequences from molecular ions between *m/z* = 1932 and 1990 were similar to each other (Table 2).

### 2.3. Sugar Inhibition of AKL

The sugar inhibition assay showed that the hemagglutinating activity of AKL was inhibited by d-galacturonic acid (0.013 mM) and moderately inhibited by d-xylose (6.25 mM). d-galactose, which is used for purification, did not show inhibition at the highest concentration tested (100 mM). This result suggested that the affinity of AKL for d-galactose was very weak and was not measurable by the above assay.

### 2.4. Primary Structure of AKL

**Figure 3:**
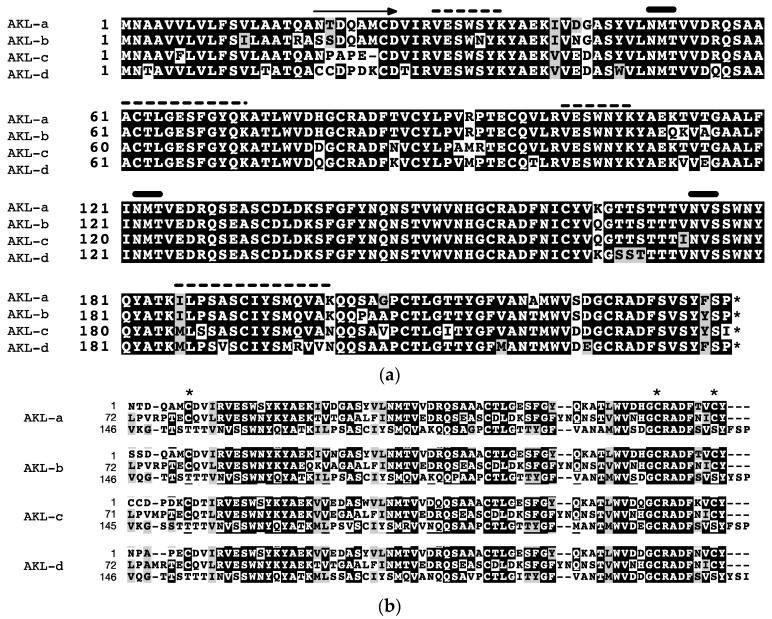
Alignment of deduced amino acid sequences of *Aplysia kurodai* lectin (AKL) cDNAs. (**a**) the amino acid sequences deduced from the AKL cDNAs. The arrow indicates the amino terminal sequence of mature AKL. The characters with black or gray background indicate identical or similar amino acids, respectively. The thick bar indicates an *N*-glycosylation site. The dotted line indicates the region with a conserved sequence as determined by *de novo* sequencing (Table 2); (**b**) the tandem repeated regions of AKLs were aligned. * indicates conserved cysteine residues.

**Figure 4:**
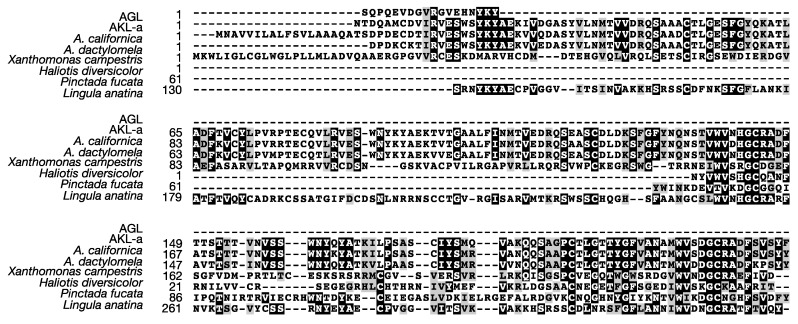
Alignment of deduced amino acid sequences of AKL-a and related proteins. The characters with black or gray background indicate identical or similar amino acids, respectively. AGL, gonad lectin of *Aplysia californica* (accession no. AAB24243). A hypothetical protein of the *A. californica* genome (accession no. XM_005101501). A galacturonic acid-binding lectin ADEL of *A. dactylomela* (accession no. ADP02966). A hypothetical protein of the *Xanthomonas campestris* genome (accession no. KIQ23628). An uncharacterized protein of *Haliotis diversicolor* (accession no. JU066540). *Pinctada fucada*, a hypothetical protein of the *P. fucada* gene pfu_aug2.0_1835.1_18561.t1 [25]. An uncharacterized protein of the *Lingula anatina* genome (accession no. XP_013393448).

We next designed primers for 5′- and 3′-RACE experiments on the basis of the *N*-terminal sequences determined above (Table 1, I to IV). Galacturonic acid-binding lectins have been reportedly obtained from the gonads and eggs of *A. depilans* and *A. californica* [21,23]. Albumen glands produce proteins in perivitelline fluid, and an egg lectin of the edible snail *Helix pomatia* was cloned from a gene extracted from the albumen gland [24]. Moreover, most of the hemagglutinating activity of egg mass was located in perivitelline fluid (data not shown), and we therefore obtained a cDNA library from the albumen gland. The 5′- and 3′-RACE PCR experiments afforded four closely related cDNAs denoted as AKL-a, -b, -c, and -d. The open reading frame of AKL-a, -b, and -d contained 711 bases, but that of AKL-c contained only 708 bases. All genes encoded an 18-residue hydrophobic signal sequence and 219- or 218-residue mature polypeptides with varying mutations (Figure 3a). More than 85% of the amino acids among those polypeptides were identical, and a well-conserved tandem repeated sequence was found (Figure 3b). The amino acid sequences estimated by mass spectral analyses, VESWSYK, VESWNYK, I/L I/L PSASX I/L YSMQVAK, and ACT I/L GESFGYQK, were also found. The deduced amino acid sequences showed 96% identity with a lectin from *A. dactylomela* [25] and a hypothetical protein predicted from *A. californica*, and some mollusks (*Haliotis diversicolor*, *Pinctada fucada*, and *Ligula anatine*). Some identity (24.4%) was found in a theoretical protein of the bacteria *Xanthomonas* (accession no. A0A0D0L3A6) (Figure 4). These proteins at 53–229 a.a. contained a protein domain called DUF3011 whose function is unknown.

**Figure 5:**
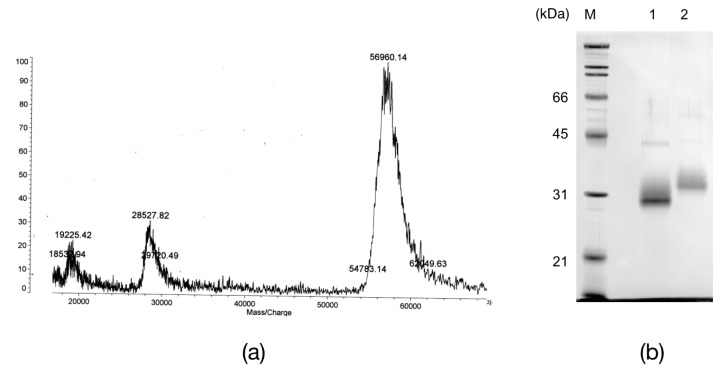
Glycopeptidase F (GPF) treatment of *Aplysia kurodai* lectin (AKL). (**a**) matrix assisted laser desorption ionization-time of flight mass spectrometry (MALDI-TOF MS) for AKL; (**b**) Sodium dodecyl sulfate-polyacrylamide gel electrophoresis of AKL (lane 2), which digested with GPF (lane 1), and molecular standards (M).

The theoretical molecular weights for AKL-a, -b, -c, and -d were 24.8, 24.4, 24.4, and 24.6 kDa respectively, differing largely from that estimated by SDS-PAGE (33.9 kDa). As the *N*-glycosylation site at position 60 was present in each polypeptide, the apparent mass difference can be attributed to sugar chains. In a MALDI-TOF MS spectrum, AKL gave a broad ion cluster centering at *m*/*z* 57,000 and *m*/*z* 28,600 (Figure 5a). If disulfide bonds assembled two monomers, the actual molecular weight of AKL would be 3800–4200 Da higher than that of the largest theoretical dimers. Treatment of AKL with *N*-glycopeptidase F resulted in a shift of the protein band from 33.9 kDa to 30.4 kDa (Figure 5b). The difference of 3500 Da corresponds well to that of the deduced molecular weight of the subunits (24.4–24.8 kDa) and that determined by MALDI-TOF MS (28,600) (Figure 5a). These results together suggested that AKL likely forms a covalent dimer, and that the protomer contains at least one sugar chain.

### 2.5. Effects of an AKL Inhibitor on Larval Morphology

**Figure 6:**
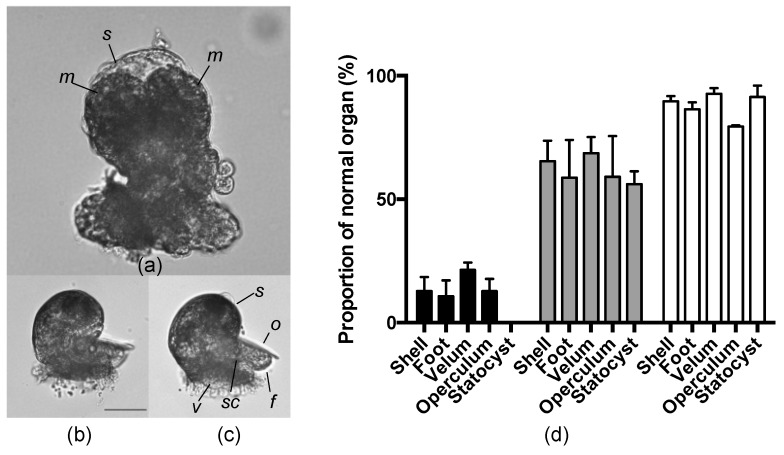
Effect of uronic acids on the development of *Aplysia kurodai* larvae. Light micrographs of veliger larvae obtained from egg string treated with 50 mM GalU (**a**, *m*: macromeres, *s*: shell), 50 mM GlcU (**b**), or filtrated sea water (FSW) without sugars (**c**, *f*: foot, *o*: operculum, *v*: velum, *s*: shell, *sc*: statolith). (**d**) Proportion of normal organs after treatment with GalU (black bars), GlcU (gray bars), and negative control (white bars).

We next analyzed the effects of AKL on larval development. Although the eggs of *A. kurodai* were surrounded by a gelatinous capsule, a small anionic dye bromophenol blue (molecular mass 670) was able to enter the perivitelline fluid (data not shown), suggesting that a small anionic sugar such as GalU (Mw 194.14) could also pass through the gelatinous membrane. As GalU strongly inhibited the hemagglutinating activity of AKL, we observed the development of *A. kurodai* eggs in the presence of GalU. Deformation in the developing embryo and larvae were observed in the group treated with GalU in a concentration dependent manner. When GalU (6.25–50 mM) was added to the egg mass, significant deformation was observed in the groups that received more than 25 mM GalU. Macromeres remained undeveloped even at seven days after the egg deposition, statocysts and an operculum were lost, and the shells were deformed (Figure 6a). The negative control d-glucuronic acid (GlcU) showed nearly no effect on egg development at the highest concentration tested (50 mM) (Figure 6b,c). The proportion of normal organs in larvae after GalU treatment was significantly decreased compared with that in the larvae without the sugar treatment. The proportion of normal organs in the group treated with GlcU was also significantly decreased, but to a lesser degree than in the GalU group (Figure 6d). These results were reproducible in another set of experiments using eggs collected on different dates. These results therefore indicated that external GalU added to the encapsulated eggs reached the egg and impacted larval development.

## 3. Discussion

We examined the egg-borne lectins of the sea hare *A. kurodai* regarding their primary structure and functions in egg development. The AKLs were previously purified, and its general properties and molecular mass were reported [12,19,20]. The AKL purified in the present study is likely be identical to that reported by Kawsar et al. [19], as their molecular mass of the subunit and sugar inhibition profile were similar to the present protein. In fact, galacturonic acid-binding lectins purified from the gonad of *A. depilans* and *A. californica* [21,23] are likely to be AKL homologues, as many *A. californica* genes homologous to AKL have been deposited in the GenBank database. Recently, an GalU-binding lectin whose amino acid sequence was closely related to AKL was reported from *A. dactylomela* egg mass [26]. Thus, it is reasonable to assume that egg lectins of the AKL class are widely distributed in genera of *Aplysia*. We thus propose that the egg lectins of *Aplysia* sharing similar amino acid sequences to AKL be called “AKL family” lectins. AKL-like proteins were also found in other marine mollusks (*H. diversicolor* and *P. fucada*); although the sequence similarity was limited to only part of the whole sequence and the distributions are unknown, they can also be classified as AKL family lectins. Therefore, the presence of these lectins in eggs needs to be examined.

We determined the amino acid sequences for four isolectins (AKL-a–d) on the basis of the nucleotide sequences of the corresponding cDNAs. Our 2DE experiments revealed that at least four polypeptides were present in the one band cutout from the SDS-PAGE. Furthermore, we noted that several *N*-terminal amino acids determined by Edman degradation differed from those deduced from cDNAs. These results suggested the highly variable nature of the lectin. As the animal used for AKL purification was a different individual from that used for cDNA cloning, the amino acid sequences of these animals likely differed, as well as in the individual level. In many cases, invertebrate lectins are present in multiple forms, that is, variable amino acid sequences are found within the lectin of the same organisms. This phenomenon might be a reflection of their plausible functional origin and innate immune system [27]. The overall motif of AKL with three tandem-repeated regions was conserved among the isoforms (Figure 4b). These repeated regions were likely to be carbohydrate recognition domains, as seen in other lectins with repeated domains [9,28,29]. Each repeated domain of AKL shared an amino acid sequence similarity with the protein domain DUF3011, whose function is unknown but is widely spread in bacteria, including plant pathogenic species. It is therefore plausible to assume that the DUF3011 domain is responsible for carbohydrate recognition.

In the present study we observed an impact of GalU treatment on organ formation in the veliger larvae of *A. kurodai*. Organogenesis starts after formation of the endoderm, mesoderm, and ectoderm by gastrulation. A key factor in organogenesis is called an organizer. In the amphibian *X. laevis*, one of the deuterostomes, the proteins activin/nodal and noggin induce organogenesis after gastrulation [30]. However, no organizer molecule has been reported in protostomes. In the mud snail *Ilyanassa obsoleta*, removal of a macromere three-dimensional cell at the 32-cell stage embryo where the cells are descendant from the large cell resulted in deformation of larvae resulting in multiple organs such as the eye, shell, heart, intestines, statocysts, opercula, larval kidneys, and certain muscles [31]. Thus, in spiralian gastropods, platyhelminthes, and mollusks, an organizer is thought to be distributed in the macromere at the two-cell stage.

As GalU is a potent inhibitor of AKL, AKL is likely responsible for normal organ formation in *A. kurodai*. Now that we have obtained the structural information of purified AKL, we are now examining the distribution and dynamics of AKL in embryonic development, to obtain deep insight into the functions of the egg lectin in organogenesis.

## 4. Materials and Methods

### 4.1. Materials

d-galactose-Sepharose 6B gel was prepared as described previously [32]. Specimens of the sea hare *A. kurodai* were collected in Okkirai Bay, Sanriku-cho Iwate, Japan and reared in a tank with running seawater from 2004 to 2009. Freshly laid egg masses from the animals were kept frozen at −70 °C until use.

### 4.2. Isolation of A. kurodai Egg Lectin

An egg mass was homogenized twice with an equal volume of 20 mM Tris-HCl buffer, pH 8.0, containing 0.15 M NaCl, and centrifuged at 12,000× *g* for 15 min at 4 °C. The combined supernatant (800 mL) was used for the further purification of lectins. The extract (200 mL) was applied on a d-galactose-Sepharose 6B gel (39 mL) column. The gel was washed with 20 mM Tris-HCl, pH 8.0, 0.15 M NaCl until the absorbance at 280 nm reached background levels. Then the adsorbed substances were eluted with 0.2 M l-galactose in 20 mM Tris-HCl containing 0.15 M NaCl, pH 8.0. Each 10 mL fraction was collected during monitoring with UV absorbance at 280 nm and with hemagglutinating activity against rabbit erythrocytes. Fractions that showed significant absorption at 280 nm and hemagglutinating activity were combined, and then dialyzed against distilled water.

### 4.3. Hemagglutination Testing

Hemagglutination testing was carried out as described previously [32]. Briefly, 20 µL of the extract was diluted in a 96-well microtiter plate by serial two-fold dilutions using 0.15 M NaCl, 20 mM Tris-HCl, pH 8.0 as a diluent. They were added to 20 µL of 4% rabbit erythrocyte suspension. After incubating at 37 °C for 1 h, the agglutination titer of the maximum dilution giving positive agglutination was recorded. The total activity was calculated as the hemagglutinating unit (HU) by multiplying a hemagglutinating titer and a sample volume (mL). The specific activity was defined as the hemagglutinating units per mg of lectin preparation.

### 4.4. Inhibition of Hemagglutination by Sugars

Inhibition of sugars and glycoproteins against hemagglutinating activity was conducted as follows. Ten µL aliquots of the extract diluted to a titer of 8 were allowed to react with 10 µL of various concentrations of inhibitors for 1 h at room temperature. Twenty µL of 4% rabbit erythrocyte suspension was then added to the mixture, and the agglutination was measured after standing for 30 min at 37 °C. The results were expressed by the minimum concentration of the inhibitors that inhibited the hemagglutination. The following sugars (200 mM) and glycoproteins (0.25%) were used as inhibitors: l-arabinose, l-fucose, d-ribose, deoxy-d-ribose, d-xylose, d-glucose, d-galactose, l-rhamnose, d-mannose, lactose, melibiose, maltose, d-glucosamine, d-galactosamine, d-mannosamine, *N*-acetyl-d-glucosamine, *N*-acetyl-d-galactosamine, fetuin, mucin Type I from bovine submaxillary gland, and mucin Type II from porcine stomach.

### 4.5. Electrophoresis

SDS-PAGE was performed in a 15% polyacrylamide gel [33]. The gel was stained with Zinc reverse staining [34].

2DE was carried out according to the manufacturer’s protocol. AKL (21 µg) was mixed with 118 µL of rehydration solution (8 M urea, 0.5% 3-[(3-cholamidopropyl)dimethylanmonio]-propanesulphonic acid, 0.5% Bio-Lyte 3/10 (Bio-Rad, Hercules, CA, USA), 0.2% dithiothreitol, and 0.002% bromophenol blue, and the mixture was sonicated for 10 min in ice water. After centrifugation, the resultant supernatant was applied to Immobilized pH gradient (IPG) strip 3-6 (Bio-Rad). Isoelectric focusing was performed as follows: 12 h rehydration, 30 min at 500 V, 30 min at 1000 V, 1 h 40 min at 5000 V. After the focusing, the strip was equilibrated in 2% SDS, 50 mM Tris-HCl, pH 8.8, 6 M urea, 30% glycerol, 0.25% DTT for 15 min with shaking. Then, it was re-equilibrated with 2% SDS, 50 mM Tris-HCl, pH 8.8, 6 M urea, 30% glycerol, 4.5% iodoacetamide for 15 min with shaking. The strip was on the 12% polyacrylamide gel, and a second electrophoresis was performed in the same manner as the SDS-PAGE. In the case of native 2DE, the 2DE was performed without urea. The gel was stained by zinc reverse staining or Coomassie Brilliant Blue staining.

### 4.6. Protein Quantification

Protein concentrations were determined by BCA protein assay kit (Thermo Fisher Scientific, Rockford, IL, USA) with bovine serum albumin as a standard.

### 4.7. Amino Acid Sequence Determination

The *N*-terminal amino acid sequences of the purified lectin and the subunits electroblotted onto a polyvinylidene difluoride membrane (FluoroTrans, PALL, Port Washington, NY, USA) after SDS-PAGE were analyzed by automated Edman degradation using a Shimadzu gas-phase PPSQ-21A sequencer (Kyoto, Japan).

The internal amino acid sequences were determined by *de novo* sequencing using MALDI-TOF MS spectrometry Autoflex III (Bruker Daltonics, Bremen, Germany) following our previous protocol [35]. Briefly, after reductive alkylation, 1.0 µg of AKL was electrophoresed by SDS-PAGE. The band of AKL was cut and digested with 0.1 µg trypsin (proteomics grade, Sigma-Aldrich, St. Louis, MO, USA). The digested peptides were extracted, and then reacted with *o*-methylisourea and sulfophenylisothiocyanate [36,37]. The peptides were purified with ZipTip C18 (Millipore, Bedford, MA, USA), and the eluate was analyzed with the MALDI-TOF-TOF Autoflex III in “Lift” mode. The partial amino acid sequences were manually assigned using an open source mass spectrometry tool mMass [38].

### 4.8. Molecular Mass Determination of AKL

The mass determination was performed using a two-layer method with some modification [39]. Sinapinic acid in acetone (10 mg/mL) was put on to a MALDI plate with 96 targets, and dried to form crystals. After drying, 1 µL of 1 mg/mL of AKL was mixed with 10 mg/mL sinapinic acid in 75% MeCN and 0.1% TFA, and placed on to the crystal and dried. The mass was determined by AXIMA CFR plus (Shimadzu Corp., Kyoto, Japan) in linear mode. Bovine serum albumin (Sigma, A4378) and horse heart myoglobin (Sigma, M-1882) were used as molecular mass standards.

### 4.9. Deglycosylation of AKL

AKL was digested with *N*-glycopeptidase F at 37 °C overnight (Takara Bio, Shiga, Japan) according to the manufacturer’s protocol in a denaturing condition. AKL (22.5 µg) was dissolved with 7.5 µL of 0.25% SDS, 0.38% 2-mercaptoethanol, 0.25 M Tris-HCl, pH 6.8 and boiled for 3 min. After addition of Nonidet P-40 with a final concentration of 1.7%, the solution was mixed with 1 µL of *N*-glycopeptidase F at 37 °C for 40 h, and then analyzed with SDS-PAGE.

### 4.10. Preparation of cDNA

Total RNA from the albumen gland was prepared using an RNA isolation kit (Gentra Systems, Minneapolis, MN, USA) according to the manufacturer’s protocol. mRNA was purified with a BioMag Poly (A)^+^ RNA purification kit (GE Healthcare, Uppsala, Sweden). cDNA synthesis was performed with a BD SMART RACE cDNA amplification kit (Takara Bio, Shiga, Japan).

### 4.11. Determination of the cDNA of AKLs

Cloning of AKL cDNAs was performed by the RACE method using a BD SMART RACE cDNA amplification kit. Degenerated primer AKLp1 (5′-CAR GAY CAR GCN ATG TGY GAY GE-3′) was synthesized on the basis of the amino-terminal amino acid sequences (QDQAMCDV). 3′ RACE with AKLp1 was performed using the DNA polymerase Advantage 2 Polymerase Mix (Takara Bio). The amplified DNAs were subjected to agarose gel electrophoresis and were purified with the QIAEX II DNA purification kit (QIAGEN, Hilden, Germany). The fragments were cloned into a vector pCR4 (Invitrogen, Carlsbad, CA, USA) and sequenced. To determine the 5’ terminal of AKL cDNAs, 5′ cDNAs were amplified using the AKLp2 (5′-GCC GAT CTA CGA CCG TCA CGA CCG TCA TGT TGA GG-3′) with the 5′-RACE method. AKLp3 (5′-CAY ATY TCT GAC GCT GCA MMA AGT YC-3′) and SMART II A Oligonucleotide were used to amplify the PCR products containing the whole translated region. All PCR conditions were as follows: (94 °C for 30 s, 70 °C for 30 s, and 72 °C for 60 s) for five cycles, (94 °C for 30 s, 68 °C for 30 s, and 72 °C for 60 s) for five cycles, and (94 °C for 30 s, 66 °C for 30 s, and 72 °C for 60 s) for 15–20 cycles.

The nucleotide sequence obtained has been deposited in the DNA Data Bank of Japan, nucleotide sequence database (accession no. AB968309, AB968310, AB968311, and AB968312). The database search and conserved domain search were performed using BLAST at the National Center for Biotechnology Information server [40,41].

cDNAs were translated and aligned with homologous genes with Clustal Omega [42,43], and these were depicted with BoxShade server [44].

### 4.12. Effects of Uronic Acids on Larvae at Different Embryonic Stages

A solution of GalU and GlcU (0.4 M) was adjusted to pH 8 by adding diluted NaOH. The sugar was added to filtrated sea water (FSW) at a final concentration of 50 mM. To the FSW, 1 cm of egg string from one individual was added and cultured for 7 days. Three egg strings were placed in a microtube, 1 mL of boiled water was added to inhibit body shrinkage of larvae, and the strings were then fixed with 2% paraformaldehyde. The gelatinous membrane was cut, and the larvae were collected by centrifugation. The larvae were photographed under a microscope. Using microscopy, 100 larvae from each treated group were observed regarding the morphology of the shell, foot, velum, operculum, and statocysts. The proportions of abnormal morphology were calculated.

### 4.13. Statistical Analysis

The data were analyzed by two-way ANOVA using GraphPad Prism version 6.0 g for Macintosh (GraphPad Software, La Jolla, CA, USA, www.graphpad.com). Logistic regression was used to evaluate the effect of timing of carbohydrate addition on embryonic morphogenesis.

## 5. Conclusions

In conclusion, we purified a GalU-binding lectin (AKL), and cloned the gene encoding the amino acid sequence of AKL. AKLs belonged to a protein family with a DUF3011 domain. As the present study is the first to unveil the possible function of DUF3011 family proteins, the function of bacterial proteins within the family can now be investigated regarding carbohydrate recognition. The lectin seems to be distributed widely in sea hare species and to be closely involved in organ formation in early developmental stages; the detailed function, mechanisms, and distribution in a wider range of invertebrate eggs are of great interest.

## Figures and Tables

**Table 1:** Amino terminal sequences of each spots of two-dimensional native polyacrylamide gel electrophoresis.

Sample	Amino Acid Sequence
SDS	N/S Q/P G/D D/P A/E M/D T/D V/D G/V I/V
I	S G D Q A M C D V I VESWNYKXA
II	N Q D Q A M C D V I VESXNYKXA
III	N Q D Q A M C D V I VESWXYKYA
IV	N Q D Q A C D V I VES

SDS indicates a band separated by sodium dodecyl sulfate-polyacrylamide gel electrophoresis. I, II, III, and IV indicate spots separated by two-dimensional electrophoresis under native conditions. These spots were named in the smaller order of the relative molecular mass (*M*r). X indicates an undetermined amino acid.

**Table 2:** Amino acid sequences obtained from trypsin digested fragments of *Aplysia kurodai* lectin AKL.

*m/z*	Amino Acid Sequence
1155	VESWSYK
1182	VESWNYK
1932	T I/L W HPXQYAK
1939	I/L I/L PSASX I/L YSMQVAK
1941	T I/L WSFGYQK
1975	ACT I/L GESFGYQK
1989	T I/L GESFGYQK
1990	T I/L WSFGYQK

X indicates undetermined amino acid. I/L indicates isoleucine or leucine.
